# Mechanisms of Enhanced Low-Temperature Lignocellulose Degradation by an ARTP-Induced *Paenarthrobacter nitroguajacolicus* Mutant: Physicochemical Characterization, Comparative Genomic Analysis, and Transcriptional Expression Profile Verification

**DOI:** 10.3390/microorganisms14040728

**Published:** 2026-03-24

**Authors:** Tianjiao Li, Yaowei Chi, Doudou Jin, Xianzhong Ma, Mengke He, Yibing Zhao, Shaohua Chu, Shunping Zhang, Pei Zhou, Dan Zhang

**Affiliations:** 1 School of Agriculture and Biology, Shanghai Jiaotong University, Shanghai 200240, China; 2 Key Laboratory of Urban Agriculture, Ministry of Agriculture and Rural Affairs, Shanghai 200240, China; 3 Shanghai Yangtze River Delta Eco-Environmental Change and Management Observation and Research Station, Ministry of Science and Technology, Ministry of Education, Shanghai 200240, China; 4 Shanghai Urban Forest Ecosystem Research Station, National Forestry and Grassland Administration, Shanghai 200240, China; 5 Chongming Agricultural Environment Field Science Observation and Research Station, Ministry of Agriculture and Rural Affairs, Shanghai 200240, China; 6 Inner Mongolia Research Institute, Shanghai Jiaotong University, Hohhot 010052, China

**Keywords:** low temperature, straw biodegradation, cellulolytic bacterium, ARTP mutagenesis, comparative genomics

## Abstract

The prolonged low temperature in cold regions significantly inhibits the initiation of straw composting and lignocellulose degradation, thereby restricting straw resource utilization. In this study, 24 cellulose-degrading strains capable of stable growth under low-temperature conditions were screened. Based on multiple indicators, including carboxymethyl cellulase (CMCase) activity, strain LDT1 was identified as the best-performing isolate under low-temperature conditions and as *Paenarthrobacter nitroguajacolicus*. Subsequently, an efficient mutant strain, LDT1-8, was obtained through atmospheric and room-temperature plasma mutagenesis. The CMCase activity of LDT1-8 at 10 °C increased to 74.25 U/mL, representing a 21.72% increase compared to the wild-type strain. In a straw degradation system at 10 °C, LDT1-8 significantly accelerated early-stage degradation kinetics, with straw degradation rates at 3 and 6 d being 72.72% and 38.15% higher than those of the wild-type strain, respectively. Multi-enzyme profiling further indicated enhanced activities of multiple lignocellulose-degrading enzymes at low temperatures, accompanied by a partial shift in the optimal temperature of some enzymes (e.g., laccase) toward lower temperatures. Whole-genome sequencing revealed increased gene numbers related to energy, amino acid, and lipid metabolism in LDT1-8. Comparative genomic analysis suggested that mutations were mainly enriched in regulatory regions, accompanied by local structural variations. Transcriptional analyses further verified the coordinated upregulation of genes involved in cellulose and hemicellulose degradation, cold adaptation, and transcriptional and protein homeostasis processes in LDT1-8. Overall, this study provides an efficient microbial resource and a mechanistic basis for straw bioconversion in cold regions.

## 1. Introduction

Agricultural straw is one of the most abundant renewable biomass resources worldwide and plays an important role in the sustainable development of agriculture and bioenergy systems [1]. However, large quantities of straw are still improperly managed or directly burned, leading to resource waste and serious environmental pollution [2]. Composting and biological conversion are regarded as effective approaches for straw valorization, yet their practical implementation remains challenging, particularly in cold and high-latitude regions where low ambient temperatures prevail.

Low temperature significantly constrains microbial activity and enzymatic reactions during straw composting. As a result, composting systems often show slow initiation, prolonged stabilization periods, and reduced degradation efficiency. In cold regions, composting systems often fail to rapidly enter an efficient thermophilic phase, which further limits large-scale application and operational stability [3]. These constraints highlight the urgent need for biological strategies that function effectively at low temperatures [4].

Cold-adapted and cold-tolerant microorganisms have been proposed as promising solutions for enhancing lignocellulose degradation at low temperatures. Several studies have reported the isolation of cold-adapted cellulolytic microorganisms from diverse environments, including soils, composts, and polar regions [4,5,6]. However, most reported cold-tolerant cellulolytic strains show limited catalytic efficiency and slow degradation initiation under low-temperature conditions, which restricts their practical application. Moreover, the regulatory mechanisms underlying low-temperature lignocellulose degradation and performance enhancement in these microorganisms remain poorly understood.

Strain improvement through mutagenesis has been widely employed to enhance microbial performance, among which atmospheric and room-temperature plasma (ARTP) mutagenesis has emerged as a powerful and efficient tool due to its high mutation efficiency, short treatment time, and minimal secondary pollution [7]. ARTP mutagenesis has been successfully applied to improve microbial traits such as enzyme production and stress tolerance [8]. In parallel, advances in whole-genome sequencing and comparative genomics enable systematic exploration of mutation landscapes and regulatory changes associated with phenotypic improvement [9]. Nevertheless, the application of ARTP mutagenesis to improve cold-tolerant cellulolytic bacteria, particularly with an emphasis on low-temperature lignocellulose degradation and its regulatory basis, remains largely unexplored.

To address these bottlenecks, this study integrates functional screening, ARTP-based strain improvement, and genome-informed analysis. Specifically, this work (i) isolates a cold-tolerant cellulolytic bacterium capable of degrading lignocellulose at 10 °C, (ii) improves its degradation performance through ARTP mutagenesis, and (iii) combines comparative genomics and transcriptional analysis to explore the potential regulatory basis underlying enhanced low-temperature lignocellulose degradation.

## 2. Materials and Methods

### 2.1. Screening of Cold-Tolerant Cellulose-Degrading Bacteria

Soil samples were collected from a long-term straw composting site in Sijiazi Village, Yong’an Town, Tuquan County, Hinggan League, Inner Mongolia (121°35′10″ E, 45°45′37″ N). The samples were transported to the laboratory at 4 °C. A soil suspension was prepared, serially diluted, and spread onto a selective screening medium containing carboxymethyl cellulose sodium (CMC-Na) as the sole carbon source. Plates were incubated at 10 °C for 7–15 days. Colonies that appeared were purified and subsequently inoculated into filter paper degradation medium, straw degradation medium, and CMC-Na–Congo red agar plates. A detailed formulation of the media used is provided in the Supplementary File. All cultures were incubated at 10 °C for 7–15 days under constant conditions, and straw degradation, filter paper degradation, and relative enzyme activity (A) were recorded [10,11]. CMCase activity was determined using a carboxymethyl cellulase assay kit (Shanghai Yuanye Bio-technology Co., Ltd., Shanghai, China), according to the manufacturer’s instructions. One unit (U) of CMCase activity was defined as the amount of enzyme required to release 1 μmol of reducing sugar per minute under the assay conditions. Detailed procedures are provided in the Supplementary File.

The relative enzyme activity of CMC-Na–Congo red agar plates was calculated using Equation (1):

(1) $$A=\frac{\mathrm{Diameter}\;\mathrm{of}\;\mathrm{hydrolytic}\;\mathrm{zone}\;(\mathrm{mm})\;}{\mathrm{Colony}\;\mathrm{diameter}\;(\mathrm{mm})\;}$$

### 2.2. Morphological Observation, Physiological and Biochemical Characterization, and Species Identification

The colony morphology of the isolates, including color, shape, size, and opacity, was carefully examined. Physiological and biochemical properties of the strain were characterized using a series of standard assays, including Gram staining, catalase activity, nitrate reduction, and oxidase tests, etc. Commercial micro-biochemical reaction tubes (Hangzhou Binhe Microbial Reagents Co., Ltd., Hangzhou, China) were used according to the manufacturer’s instructions. Briefly, bacterial suspensions were inoculated into the reaction tubes and incubated under appropriate conditions, and the results were determined based on visible color changes indicating positive or negative reactions. The identification and interpretation of physiological and biochemical characteristics were performed with reference to Bergey’s Manual of Systematic Bacteriology and the Manual for the Identification of Common Bacteria. Genomic DNA was extracted using the MiniBEST Bacteria Genomic DNA Extraction Kit Ver. 3.0 (TaKaRa, Shiga, Japan). The full-length 16S rRNA gene was amplified by PCR with the universal primers 27F (5′-AGAGTTTGATCCTGGCTCAG-3′) and 1492R (5′-GGTTACCTTGTTACGACTT-3′) [12]. The PCR products were sequenced by Personalbio Co., Ltd. (Shanghai, China). The resulting sequences were compared against the NCBI BLAST database (https://blast.ncbi.nlm.nih.gov/Blast.cgi), and a phylogenetic tree was constructed using the Neighbor-Joining method.

### 2.3. Mutagenesis of Cold-Tolerant Cellulose-Degrading Strains by ARTP and Evaluation of Mutagenic Effects

The activated strain was inoculated into LB liquid medium (100 mL in a 250 mL flask) and cultured at 30 °C with shaking at 200 rpm until reaching the exponential growth phase. Once the culture reached the logarithmic phase, 1 mL of bacterial suspension was collected and centrifuged at 6000 rpm for 5 min to harvest the cells. The cell pellet was washed with a sterile solution to remove residual medium and impurities. The washed cells were then resuspended and diluted in sterile solution to an OD600 of 0.8 to obtain the required cell suspension. Prior to mutagenesis, the ARTP (Atmospheric and Room Temperature Plasma) system (Yuanqing Tianmu Biotechnology Co., Ltd., Wuxi, China) was sterilized under UV light for 30 min. Sterile forceps were used in a biosafety cabinet to place glass slides into the designated grooves of the ARTP operating chamber. A 1 mL sterile solution in an EP tube was positioned directly beneath the groove and secured in the tube holder. The ARTP parameters were set as follows: discharge power at 120 W, helium gas flow rate at 10 SLM, treatment distance of 2 mm, and cooling water circulation at 20 °C. Exposure times were set to 0, 15, 30, 45, 60, 90, 120, and 150 s [7,13]. Immediately after treatment, the EP tubes were removed and vortexed for 1 min to resuspend cells.

The treated bacterial suspension was serially diluted to 10^3^–10^7^ fold using sterile saline. A volume of 100 μL from each dilution was spread onto LB agar plates. Each treatment was performed in triplicate. Plates were incubated at 30 °C for 72 h. An untreated bacterial suspension served as the control. During sampling, the suspensions were mixed thoroughly to ensure cell uniformity. After incubation, colonies were counted and the mortality rate was calculated using the following Equation (2):

(2) $$\mathrm{Mortality}\;\mathrm{rate}=\frac{N_{0}-N_{1}}{N_{0}}\times 100\%,$$

where N_0_ is the number of colonies in the control group, and N_1_ is the number in the treatment group.

Approximately 100 individual colonies were selected from the treatment group with the appropriate mutation time and inoculated into 96-well plates at a 2% inoculation ratio. The OD_600_ values were measured after 24 h of incubation, and superior mutants (OD_600_ ≥ 0.8) were further screened for CMCase activity. Among these candidates, strain LDT1-8 exhibited the highest CMCase activity and was therefore selected for subsequent analyses. To verify the genetic stability of the LDT1-8 strain’s enzyme-producing ability, the strain was continuously streaked on LB solid medium for 6 generations and then subjected to shake flask fermentation to determine the activity of CMCase enzyme.

### 2.4. SEM Observation and Cellulose Degrading Enzyme Spectrum Analysis of LDT1-8 Strain

The impact of atmospheric and room temperature plasma (ARTP) mutagenesis on strain performance was assessed by combining morphological and functional analyses. The cell morphology before and after the mutation was observed using a scanning electron microscope (SEM), following the previous method [14]. Enzymatic assays were performed to evaluate changes in the activity of multiple hydrolases. CMCase, xylanase, pectinase, laccase, β-glucosidase, endoglucanase, and filter paperase (FPase) activities were measured using commercial assay kits from Shanghai Yuanye Bio-Technology Co., Ltd. (China) and Shanghai Meirui Biochemical Technology Co., Ltd. (Shanghai, China). Enzyme activities were expressed as U/mL. Moreover, activities were measured across a broad temperature gradient (4, 10, 15, 20, 30, and 40 °C) to assess the thermal adaptability of the strains. In addition, bacterial suspensions were inoculated into straw degradation medium (Supplementary File) at an inoculum volume of 2% (*v*/*v*) and incubated at 10 °C and 30 °C with shaking at 200 rpm. The residual straw weight in the medium was measured on days 0, 3, 6, 9, 12, and 15. A deionized water control test was also conducted to assess abiotic weight loss.

### 2.5. Whole-Genome Sequencing and Comparative Genomic Analysis of Strains LDT1 and LDT1-8

The genomes of strains LDT1 and LDT1-8 were sequenced and assembled de novo by Majorbio Bio-Pharm Technology Co., Ltd. (Shanghai, China), using a hybrid strategy that integrated Illumina HiSeq (second-generation) and PacBio (third-generation) sequencing platforms. More than 100-fold coverage was achieved with both Illumina and PacBio reads, ensuring high-quality assemblies. Detailed analysis steps can be found in the Supplementary File. Whole-genome sequence data of LDT1 and LDT1-8 have been deposited in GenBank under accession numbers CP199916 and JBRACU000000000.

### 2.6. Transcriptional Expression Profile of Functional Genes

To verify whether ARTP-induced mutations resulted in transcriptional changes related to low-temperature cellulolysis, quantitative real-time PCR (qRT-PCR) was performed to compare gene expression profiles between the wild-type strain LDT1 and the mutant strain LDT1-8 at 10 °C and 30 °C. After RNA extraction and cDNA synthesis, the transcription levels of key genes involved in cellulose/hemicellulose degradation, transcriptional regulation, and cold adaptation were quantified. The 16S rRNA gene was used as the internal reference gene for normalization. The gene relative expression levels were calculated using the 2^−ΔΔCt^ method [15]. All qRT-PCR assays were performed with three independent biological replicates, each with three technical replicates. Primer specificity was confirmed by agarose gel electrophoresis and melting curve analysis, showing single specific amplification products. Detailed experimental procedures are provided in the Supplementary File.

### 2.7. Statistical Analysis

All experiments were conducted with three biological replicates. Data normality and homogeneity of variance were assessed using the Shapiro–Wilk and Levene tests, respectively. Statistical analyses were conducted using one-way analysis of variance (ANOVA) followed by Tukey’s multiple comparison test, as well as Student’s *t*-test (SPSS v26.0) [16]. Statistical significance was considered at *p* < 0.05. Coding sequences (CDSs) were predicted with Glimmer v3.02. Functional annotation of predicted genes was performed against the COG database and the Kyoto Encyclopedia of Genes and Genomes (KEGG). Variant effect classification was carried out using SnpEff. Figures and tables were generated with Origin 2024 and R 4.4.2.

## 3. Results and Discussion

### 3.1. Screening of Cold-Tolerant Cellulolytic Bacteria

Using sodium carboxymethyl cellulose (CMC-Na) as the sole carbon source, soil-derived microbial consortia were enriched and screened at 10 °C, resulting in the isolation of 24 bacterial strains capable of sustained growth under low-temperature conditions. These isolates were preliminarily identified as cellulolytic bacteria based on their ability to utilize CMC-Na. To systematically evaluate their cellulose-degrading potential, a stepwise multi-criteria screening strategy was applied. First, extracellular cellulase secretion was assessed using CMC-Congo red plate assays, and relative hydrolysis activity was recorded as a semi-quantitative indicator (Table S1). Although several isolates (e.g., LDF4 and LDF6) exhibited larger hydrolysis halos, this trait did not consistently correlate with subsequent degradation performance on insoluble cellulose substrates.

Next, filter paper disintegration assays were conducted to evaluate the degradation of crystalline cellulose (Table S1). Strain LDT1 exhibited effective filter paper disintegration (++), indicating strong hydrolytic activity toward structurally ordered cellulose. In contrast, some isolates displayed weak or no visible degradation despite pronounced halo formation on CMC plates. To further assess performance on complex lignocellulosic substrates, straw disintegration assays were performed (Table S1). Strain LDT1 showed clear straw degradation (++), demonstrating its ability to degrade heterogeneous plant biomass under low-temperature conditions. Notably, many isolates exhibited substrate-specific degradation preferences, performing well on either filter paper or straw but not both.

**Figure 1:**
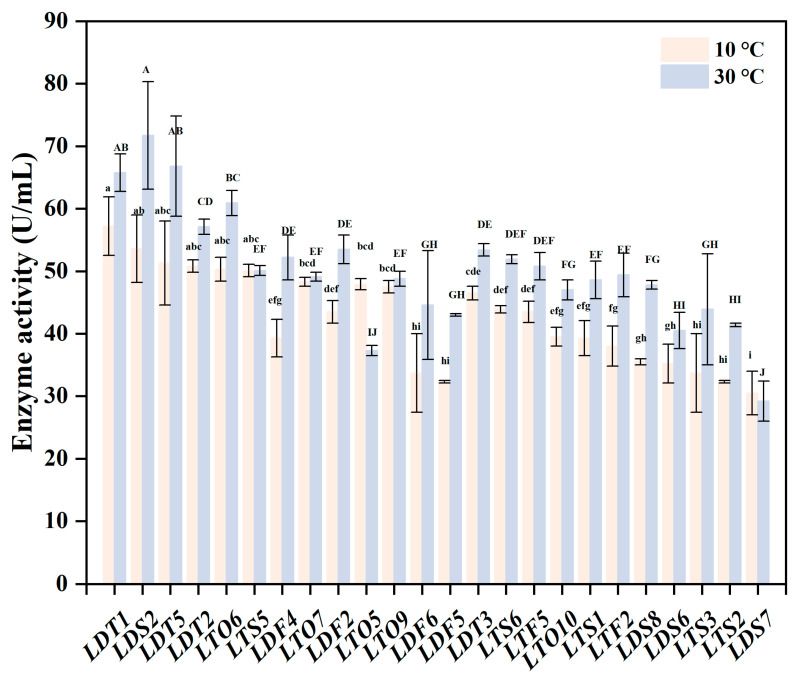
Carboxymethyl cellulase activity of different selected strains under low-temperature and normal-temperature conditions. The data are presented as mean ± SD (*n* = 3). Different letters represent significant differences between groups according to Tukey’s multiple comparison (*p* < 0.05); the comparison at 10 °C and 30 °C is represented by lowercase and uppercase letters, respectively.

Finally, CMCase activity of all isolates was quantitatively measured at both 10 °C and 30 °C (Figure 1) to validate enzymatic performance. At 10 °C, strain LDT1 exhibited the highest CMCase activity (57.20 U/mL), indicating strong intrinsic cellulolytic potential under low-temperature conditions. Although some strains showed comparable or higher CMCase activity at 30 °C, they did not maintain superior performance at 10 °C, which is more relevant for the targeted application. Overall, strain selection was based on a stepwise evaluation integrating extracellular enzyme secretion, degradation performance on both crystalline and complex cellulose substrates, and quantitative enzyme activity under low-temperature conditions. Strain LDT1 consistently ranked among the top-performing strains across all evaluation stages and exhibited the most balanced and robust cellulolytic performance. Therefore, it was selected for subsequent mutagenesis and mechanistic investigation.

### 3.2. Physiological and Biochemical Characterization and Identification of Strain LDT1

The plate streaking test showed that the LDT1 strain formed circular, smooth, moist, light yellow and opaque colonies with a diameter of 2–3 mm. The Gram staining method, combined with an optical microscope (400×) observation results, indicated that the strain LDT1 was a Gram-positive strain (Figure S1).

The physiological and biochemical characteristics of strain LDT1 are presented in Table S2. The strain exhibited metabolic versatility, effectively utilizing glucose and citrate while testing negative for common disaccharides. Notably, LDT1 showed a positive reaction to polyphenolic substrates (e.g., gossypol), indicating a robust tolerance to complex phenolic compounds. This trait is physiologically significant because phenolic byproducts released during lignin depolymerization often inhibit microbial growth. The ability to metabolize or tolerate such aromatic structures is a hallmark of efficient lignocellulose-degrading bacteria [17,18]. Furthermore, the strain exhibited active nitrogen metabolism (urease and nitrate reduction positive), suggesting an adaptability to nitrogen-limited lignocellulosic substrates.

**Figure 2:**
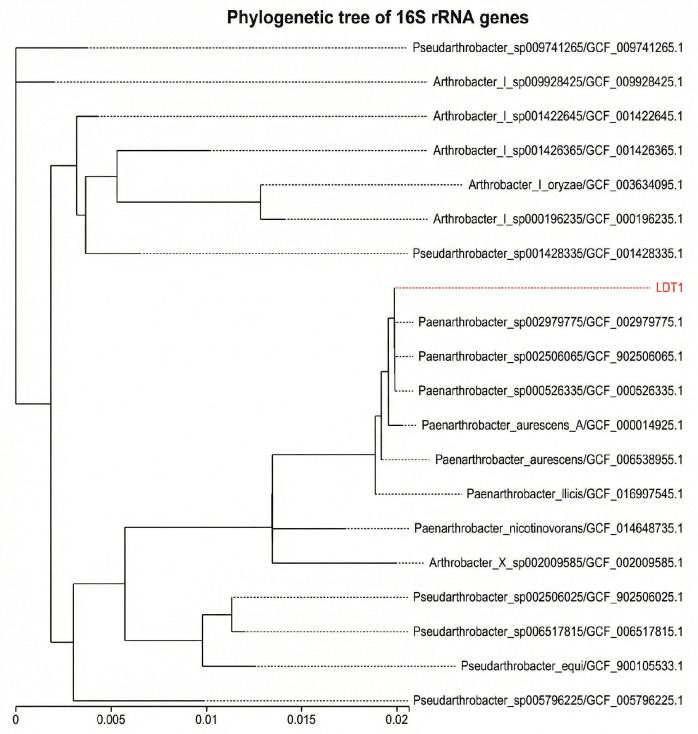
The phylogenetic tree of LDT1 based on the 16S rRNA gene. The red font represents the tested strain.

These biochemical features align with the phenotypic traits of the genus *Paenarthrobacter* (formerly distinct members of *Arthrobacter*). Combined with the 16S rRNA gene sequencing results, strain LDT1 was definitively identified as *Paenarthrobacter nitroguajacolicus* (100% identity to the reference genome assembly GCF_002979775.1) (Figure 2). This taxonomic classification strongly supports our finding of its potential for straw degradation. *Paenarthrobacter* are genomically equipped with extensive pathways for the catabolism of recalcitrant aromatic compounds, including lignin-derived monomers such as guaiacol and protocatechuate [19]. Moreover, members of this genus are widely recognized as psychrotolerant bacteria, capable of secreting cold-active enzymes (e.g., cellulases and oxidases) that maintain catalytic efficiency at low temperatures [20]. Therefore, the identification of LDT1 as *P. nitroguajacolicus*, coupled with its observed enzymatic activity at 10 °C, confirms its potential as a valuable candidate for lignocellulose bioconversion in cold climates. The strain has been deposited in the China General Microbiological Culture Collection Center (CGMCC) under accession CGMCC 31578.

### 3.3. Evaluation of ARTP Mutagenesis Effects

#### 3.3.1. Screening of Mutagenic Strains

The results showed that the mortality rate increased with increasing mutagenesis time. When the strain was treated for 90 s, the mortality rate increased rapidly to 69.70%, and then it exceeded 90% at 120 s. Therefore, a treatment time of 60 s was selected (Figure S2).

**Figure 3:**
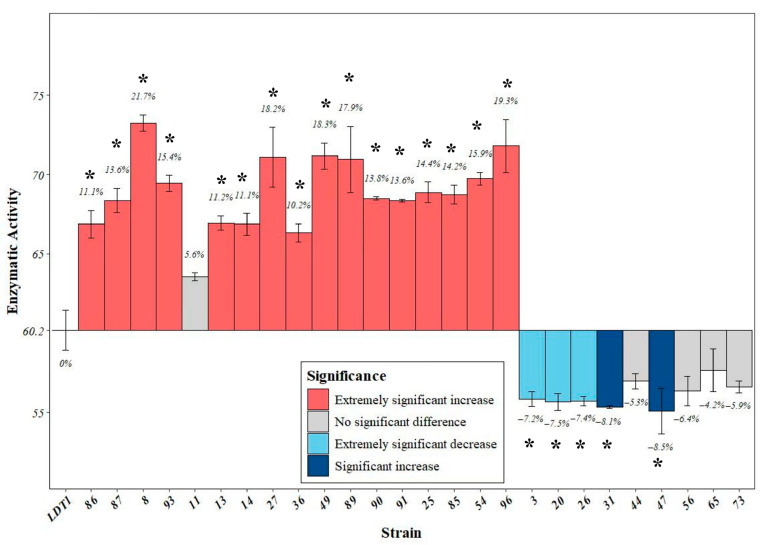
CMCase enzyme activity of ARTP mutagenesis strains. Data are presented as mean ± SD (*n* = 3). Asterisks indicate significant differences compared to the LDT1 group, as determined by Student’s *t*-test (* *p* < 0.05).

From the plates that were diluted and streaked after mutagenesis, 26 strains with good growth were randomly selected and their CMCase enzyme activity under low-temperature conditions was measured. As shown in Figure 3, 17 strains had significantly higher enzyme activity than the original strain, indicating they were positive mutant strains. Among them, the CMCase enzyme activity of the LDT1-8 strain reached up to 74.25 U/mL, which was 21.72% higher than the enzyme activity before mutagenesis. Compared with those cellulose-decomposing microorganisms with cold adaptability previously reported, for example, the CMCase activity secreted by *Penicillium chrysogenum* LTF21 at 10 °C was 19.55 U/mL, and the mutant strain LDT1-8 showed higher CMCase activity under similar temperature conditions [21]. The results of the genetic stability of CMCase enzyme activity in the mutagenized strains are shown in Figure S3. After the second generation, the CMCase enzyme activity of the LDT1-8 strain became almost stable. And the CMCase activity remained stable after six consecutive generations. Therefore, LDT1-8 was selected as the mutagenic strain for the subsequent experiments.

#### 3.3.2. Observation of the SEM Morphology of the Mutant Strain

**Figure 4:**
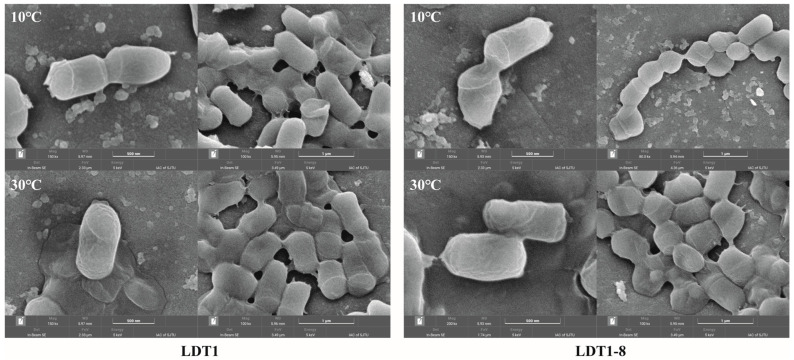
The morphological changes in the mutant strain and the wild-type strain before and after mutagenesis were observed using a scanning electron microscope.

The cell morphology of the LDT1 and LDT1-8 strains under 10 °C and 30 °C conditions was observed using a scanning electron microscope, and the results are presented in Figure 4. Under low-temperature conditions, LDT1 exhibits a shorter rod-like structure with a compact cell morphology and a relatively shorter overall length. In contrast, at normal temperatures, the LDT1 strain shows a morphological change, with the cells significantly elongated and presenting a longer rod-like shape, along with a smooth surface. The reason for this change might be that the LDT1 strain has more active metabolic activities at higher temperatures, resulting in accelerated cell growth and division, and thus, its cell morphology becomes more extended. The morphological changes in the LDT1-8 strain are also very obvious. Under low-temperature conditions, LDT1-8 presents as a shorter and relatively enlarged rod-shaped structure, with a more rounded and expanded cell shape. This may indicate that the LDT1-8 mutant strain has better adaptability to low temperatures, storing more nutrients through cell enlargement to cope with the metabolic pressure brought by the low-temperature environment. Compared to low temperatures, the cells of LDT1-8 are more enlarged at normal temperatures, and the cell expansion is more significant.

#### 3.3.3. Comparative Analysis of Enzymatic Properties Between Mutant and Wild-Type Strain

**Figure 5:**
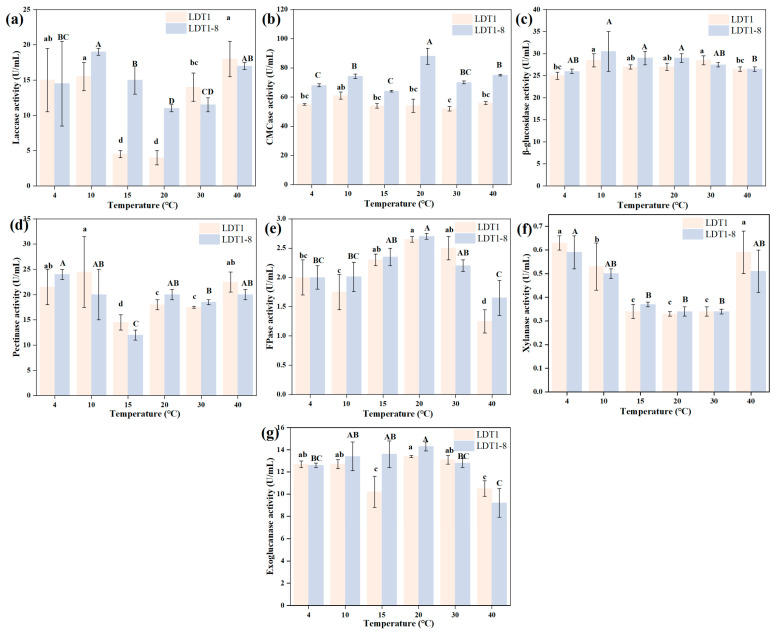
The cellulase activity spectra of mutant strains and wild-type strains at different temperatures. The data are presented as mean ± SD (*n* = 3). Different letters represent significant differences among the various temperatures according to Tukey’s multiple comparison (*p* < 0.05); the LDT1 group and the LDT1-8 group are respectively represented by lowercase and uppercase letters. (**a**) Laccase, (**b**) CMCase, (**c**) β-glucosidase, (**d**) Pectinase, (**e**) FPase, (**f**) Xylanase, (**g**) Exoglucanase.

To assess the impact of ARTP mutagenesis on the low-temperature degradation potential of the strain, we systematically compared the activities of seven key lignocellulose-degrading enzymes of the mutant strain LDT1-8 with those of the wild-type strain LDT1 at different temperatures (4–40 °C) (Figure 5). Overall, ARTP mutagenesis reshaped the enzyme system of the strain and improved its catalytic efficiency under low-temperature conditions. CMCase, as one of the rate-limiting enzymes for cellulose degradation, exhibited the most significant improvement effect in the mutant strains. The results showed that the CMCase activity of LDT1-8 was significantly higher than that of the wild type throughout the tested temperature range. The variation pattern of laccase reveals another adaptive mechanism: a shift in the optimal temperature toward lower values. The laccase activity of the wild-type strain LDT1 is only 15.51 U/mL at 10 °C, and it shows an upward trend as the temperature increases (remaining at a high level at 30 °C). In contrast, the laccase of the mutant strain LDT1-8 increases to 19.02 U/mL at 10 °C (an increase of 22.63%), and this temperature is its optimal catalytic temperature; when the temperature rises to 30 °C, its activity instead drops to 11.5 U/mL. This phenomenon of “enhanced activity at low temperatures and reduced activity at higher temperatures” conforms to the typical “activity-stability trade-off” hypothesis of cold-active enzymes [22,23]. In order to achieve greater catalytic flexibility at low temperatures, enzyme molecules often sacrifice their thermal stability. Laccase is a key enzyme involved in lignin degradation. Therefore, this characteristic makes LDT1-8 particularly suitable for overcoming the lignin barrier during the early stage of low-temperature composting. For other auxiliary enzyme systems, such as FPase, exoglucanase, and β-glucosidase, LDT1-8 exhibited varying degrees of activity enhancement at 10 °C (with the enhancement ranging from 5% to 15%). Moreover, although pectinase and xylanase showed slight fluctuations or remained stable at 10 °C, considering that the degradation of cellulose and lignin is the core rate-limiting step in the decomposition of straw, the significant enhancement of these core enzyme systems is sufficient to prove that strain LDT1-8 is an excellent mutant strain.

#### 3.3.4. Evaluation of Straw Degradation Performance at Low-Temperature

Under low-temperature conditions, a time series comparison of the straw degradation performance of the mutant strain LDT1-8 and the wild-type strain LDT1 was conducted (Figure S4). The results showed that the ARTP mutagenesis significantly accelerated the early stage of straw degradation. At 3 d of cultivation, the straw degradation rate of LDT1-8 was 19.00%, which was significantly increased by 72.72% compared to the wild-type group. At 6 d of cultivation, the degradation rate of LDT1-8 further increased to 29.04%, which was significantly increased by 38.15% compared to the wild-type group. In contrast, at 9 d and 15 d of cultivation, the straw degradation rates of the two strains gradually approached and stabilized. These results indicate that ARTP mutagenesis did not significantly increase the ultimate degradation capacity of the strain, but mainly enhanced the initiation efficiency and early advancement rate of straw degradation. Previous studies have pointed out that in the lignocellulose degradation system, the early degradation kinetics is usually limited by the initial colonization ability of microorganisms on the complex substrate surface and the rapid accumulation of exogenous degrading enzymes, rather than the final degradability ratio of the substrate [24,25]. Therefore, achieving faster early degradation under low-temperature conditions has important significance for shortening the processing cycle and improving the operational efficiency of the system.

### 3.4. Comparative Genomic Analysis of Strain LDT1 and LDT1-8

#### 3.4.1. Genomic Features

In order to elucidate the molecular mechanism underlying the enhanced low-temperature cellulose-decomposing performance of this mutant, whole-genome sequencing was conducted on the wild-type strain LDT1 and the strain LDT1-8 derived from the ARTP source. The genome sizes and GC contents of these two strains were highly similar. The genome size of LDT1 was approximately 4,406,289 base pairs (GC content of 61.96%), while that of LDT1-8 was approximately 4,374,204 base pairs (GC content of 62.00%). This indicates that the genomic compositions of the two strains are highly similar at the macroscopic level (Figure S5).

The outer ring of the CGView map shows the functional classification of different genes. The distribution of genes related to energy production and conversion (category C) and amino acid transport and metabolism (category E) in LDT1-8 is higher than that in LDT1, indicating an improvement in the metabolic efficiency of these pathways. In contrast, LDT1 shows significant differences in the distribution of genes related to cell wall/membrane/envelope biogenesis (category M) and cell motility (category N), which may reflect the differences in environmental adaptability and motility between these two strains.

#### 3.4.2. Functional Genome Annotation

##### COG Functional Classification

**Figure 6:**
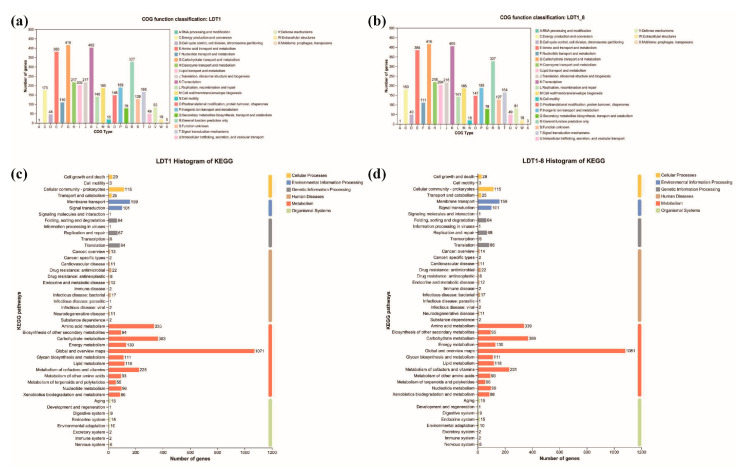
Genome COG functional classification (**a**,**b**) and KEGG pathway (**c**,**d**) analysis of wild-type (**a**,**c**) and mutant strains (**b**,**d**).

COG-based functional profiling revealed broadly similar category distributions between LDT1 and LDT1-8, with slight differences in gene counts assigned to specific functional categories. In LDT1-8, a modest increase in the number of genes annotated to categories related to energy production and conversion, cell cycle control, amino acid metabolism, lipid metabolism, and transcriptional regulation was observed (Figure 6a,b). These differences likely reflect variations in functional annotation or gene classification associated with sequence variation, rather than true gene gain. These shifts may be associated with enhanced metabolic activity under low-temperature conditions. However, no direct causal relationship can be established. No evidence of large-scale gene gain or horizontal gene transfer was detected between the two strains. These differences may result from minor sequence variations influencing gene prediction boundaries or annotation assignments in homologous regions.

Specifically, LDT1-8 annotated five more genes than LDT1 in the energy production and conversion category. The enhanced energy generation capacity is a significant characteristic requirement for the activities of microorganisms in cold environments, especially for pathways with high energy demands, such as cellulose decomposition. This indicates that an increase in gene dosage in this category can support more efficient metabolic processes under low-temperature conditions. Within Cell cycle control, cell division, and chromosome partitioning, LDT1-8 annotated 3 more genes than LDT1. Cold conditions slow biosynthesis and cell division. Improved cell-cycle regulation may therefore promote faster biomass turnover and sustained production of degradative enzymes, supporting overall catabolic performance at low temperatures [26]. For Amino acid transport and metabolism, 4 additional genes were annotated in LDT1-8. The supply of amino acids is a direct determinant of protein (enzyme) synthesis, while low temperatures often limit amino acid metabolism. Therefore, enhancing the transport and metabolism capabilities can maintain translation efficiency and increase the activity of hydrolase enzymes under low-temperature conditions. In Lipid transport and metabolism, LDT1-8 annotated 2 extra genes. The lipid components affect the fluidity and stability of the membrane, and these parameters are closely related to the function of enzymes and growth at low temperatures. Consistent with the results observed by scanning electron microscopy: at 10 °C, the rods of LDT1-8 are shorter, thicker and rounder. These changes may reflect the remodeling of the lipid pathway, which can stabilize the dynamics of the membrane and help maintain higher enzyme activity at low temperatures [27].

##### KEGG Pathway Mapping

Comparative mapping revealed noticeable differences within the Metabolism hierarchy, most prominently in Global and overview maps and Metabolism of cofactors and vitamins (Figure 6c,d). The global overview layer integrates the core metabolic nodes, including glycolysis, the tricarboxylic acid cycle, and the energy-yielding modules. The combination of genes in these modules determines the flux and energy balance of the cell. In LDT1-8, the more widespread distribution of genes at these hubs is consistent with a more robust control architecture under cold conditions [28]. Meanwhile, cofactors and vitamins play a crucial role in the catalysis, assembly and stability of enzymes. The changes in these biosynthetic pathways in LDT1-8 indicate that the supply of cofactors/vitamins has been enhanced, which may help increase the yield and activity of cellulase, thereby enhancing the fiber decomposition effect under low-temperature conditions [29]. These KEGG-level changes suggested a more complete and resilient metabolic network in LDT1-8 under cold conditions, particularly through enhancements in cofactor- and vitamin-related pathways that align with improved biodegradation potential. Based on the aforementioned analysis, the gene distributions of the two strains in core biological processes (including energy metabolism, amino acid metabolism, and carbohydrate metabolism, etc.) are roughly similar. In contrast, LDT1-8 shows significant differences from LDT1 in the categories related to transcriptional regulation, signal transduction, and membrane transport, indicating that potential changes may have occurred in metabolic regulation and environmental adaptability.

#### 3.4.3. Comparative Annotation of Carbohydrate-Active Enzymes (CAZymes)

CAZymes-based annotation indicated that both LDT1 and LDT1-8 harbor a broad repertoire of carbohydrate-active functions spanning enzymes involved in polysaccharide deconstruction and remodeling (Figure S6). Glycoside hydrolases (GHs)constituted the largest fraction, underscoring substantial potential for cellulose and hemicellulose breakdown. Glycosyl transferases (GTs), which mediate polysaccharide synthesis and modification, were also well represented, suggesting that these strains not only catabolize carbohydrates but may also contribute to cell-wall construction and remodeling. Other types of proteins that function in specific degradation pathways, such as polysaccharide lyases (PLs) and carbohydrate esterases (CEs), are also present in these pathways and are likely to support the de-esterification and β-elimination steps within complex sugar chains. The detection of auxiliary activities (AAs) and carbohydrate-binding modules (CBMs) further indicates that the synergistic oxidation effect and substrate targeting ability have been enhanced, thereby overall facilitating the effective attack on difficult-to-decompose polysaccharide structures. Relative to LDT1, LDT1-8 showed an increased representation of GHs, rising from 40.99% to 50.59% of the CAZyme complement. This shift is consistent with strengthened capacities for cellulose and hemicellulose depolymerization and may help explain the mutant’s more effective carbohydrate degradation under low-temperature conditions.

#### 3.4.4. Cold-Adaptation and Cellulolysis-Related Genes

Genomic analyses of LDT1 and its ARTP-derived mutant LDT1-8 identified a range of genes associated with cold adaptation (Tables S3 and S4). Among these, *cspA*, which encodes cold shock protein A, plays a critical role in stabilizing mRNA secondary structures, facilitating ribosome binding, and enhancing translation efficiency under low-temperature conditions [30]. Several genes encoding molecular chaperones were also detected, including *groEL*, *groES*, *dnaK*, *dnaJ*, *clpB*, and *clpC*. These chaperones are essential for maintaining protein folding and repair during cold stress, ensuring proper cellular function [31,32]. In addition, the heat shock transcription factor gene *hrcA* was identified, which regulates the expression of these chaperones through negative feedback in response to temperature fluctuations [33,34]. Genes involved in energy metabolism were also enriched in both strains. The presence of *cydA*, *cydB*, and a full set of atp genes (*atpA*–*atpI*) suggests the existence of a stable energy generation system capable of supporting metabolic activity at low temperatures [35,36,37]. This system may contribute to cold resilience by maintaining membrane stability and ensuring sufficient ATP supply [38]. In terms of oxidative stress response, several key antioxidant genes were identified. These include *katE* (encoding catalase), *trxA*, and *trxB* (components of the thioredoxin system). These genes help mitigate the accumulation of reactive oxygen species generated during cellulose degradation, reducing oxidative stress under cold-induced metabolic imbalance and thereby improving cell survival [39].

The genomes also harbored a suite of genes related to cellulose degradation (Tables S5 and S6). Notable examples include *bglB* and *bglX* (encoding β-glucosidases), which catalyze the conversion of cellooligosaccharides into glucose monomers, and *cbpA*, which encodes a cellulose-binding protein that enhances enzyme–substrate interactions. Together, these components facilitate efficient cellulose breakdown [40,41]. Genes related to hemicellulose and xylan degradation were also abundant. These include *xylA*, *xylB*, *xynB*, and *xynD* (involved in xylan hydrolysis), as well as *manA*, *manB*, and *abfA* (linked to hemicellulose degradation). Since hemicellulose and xylan are major structural polysaccharides in plant cell walls, these enzymes collectively enable broad-spectrum degradation of complex lignocellulosic materials [42,43]. The gene *gdhA*, encoding glucose dehydrogenase, was also identified and is likely involved in the further metabolism of degradation products and carbon flux regulation, contributing to energy production and biosynthetic precursor supply. Overall, both LDT1 and LDT1-8 harbor an array of functional genes associated with cold adaptation, oxidative stress resistance, carbohydrate metabolism, and polysaccharide degradation. These features indicate a coordinated metabolic strategy that enables the strains to remain active under cold conditions and efficiently decompose lignocellulosic substrates.

#### 3.4.5. Variant Annotation and Synteny Analysis

Variant calling using Snippy identified three mutation loci, two single-nucleotide polymorphisms (SNPs) and one multi-nucleotide polymorphism (MNP). Functional annotation using SnpEff indicated that these variants were located in regulatory and intergenic regions and were associated with multiple nearby genes. In total, 26 genes were potentially affected based on upstream/downstream proximity and annotation, including 12 upstream, 11 downstream, one intron, and two non-coding transcriptional exons variations (Table S7). This is because a single variant locus may fall within overlapping regulatory regions or between adjacent genes, and can therefore be annotated relative to more than one gene.

**Figure 7:**
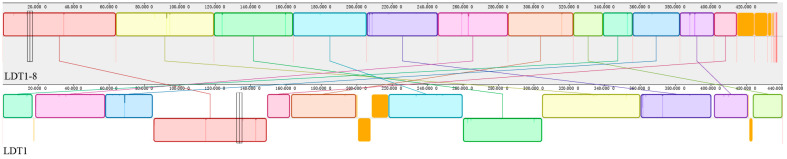
Synteny analysis between LDT1-8 and LDT1.

Genome assembly statistics for both strains are summarized in Table S8. The assembly of LDT1-8 exhibited a genome size of 4.37 Mb, scaffold N50 of 401,342 bp, sequencing depth of 278×, and completeness of 99.2%, indicating high-quality assembly. No large structural variations or horizontal gene transfer events were detected, suggesting that the observed phenotypic differences are likely associated with these limited mutations. Genome alignment with Mauve 2015 revealed a highly conserved genomic structure between the two strains, with most regions forming locally collinear blocks (LCBs). Nevertheless, inversion events were identified in specific regions, indicating localized chromosomal rearrangements. Although these structural changes did not involve large insertions or deletions, they may still influence gene expression by altering local regulatory landscapes (Figure 7). In total, 26 mutated genes were identified in LDT1-8, many of which are associated with key functional pathways such as nitrogen metabolism, aromatic amino acid biosynthesis, transcriptional regulation, peptide utilization, and oxidative stress response (Table S9). These pathways are likely to contribute to the strain’s enhanced adaptation to low temperatures.

For example, mutations in *nasC* and *narK* may improve nitrate and nitrite transport and reduction, which is beneficial for maintaining nitrogen supply under carbon-rich but nitrogen-limited composting conditions [44]. Similarly, mutations in *aroE*, *aroB*, *aroK*, and *aroC* may enhance the biosynthesis of aromatic amino acids such as tyrosine and phenylalanine, which serve as precursors for membrane components, signaling molecules, and enzyme active sites [45]. A mutation in *pepQ* may improve the recycling of short peptides, thereby supporting amino acid metabolism and providing substrates for enzyme synthesis [46,47]. Additionally, mutations identified in regulatory genes such as *lacI* and *nusB* may be associated with altered transcriptional regulation of cellulose-degrading genes. For example, *lacI* is known as a transcriptional repressor, and its mutation could potentially influence the expression of genes such as *bglB*, *bglX*, *xynB*, and *manB* [48,49]. Similarly, *nusB*, which is involved in transcriptional anti-termination, may be related to changes in mRNA stability under cold conditions [50,51]. However, these interpretations are based on genomic annotation and known gene functions, and do not establish a direct causal relationship. Further functional validation (e.g., complementation assays or promoter activity analysis) will be required to confirm these hypotheses. Therefore, the mutation–function associations described in this study should be considered as predictions based on comparative genomic analysis rather than experimentally validated functional relationships. Overall, these findings indicated that LDT1-8 accumulates a series of functionally significant mutations in multiple pathways, thereby achieving coordinated optimization of nitrogen metabolism, amino acid biosynthesis, transcriptional regulation, and enzyme expression. This gene reprogramming is likely to explain the improvement in cell decomposition efficiency in the cold composting environment.

### 3.5. Transcriptional Reprogramming Underlying Enhanced Cellulolytic Performance of LDT1-8

**Figure 8:**
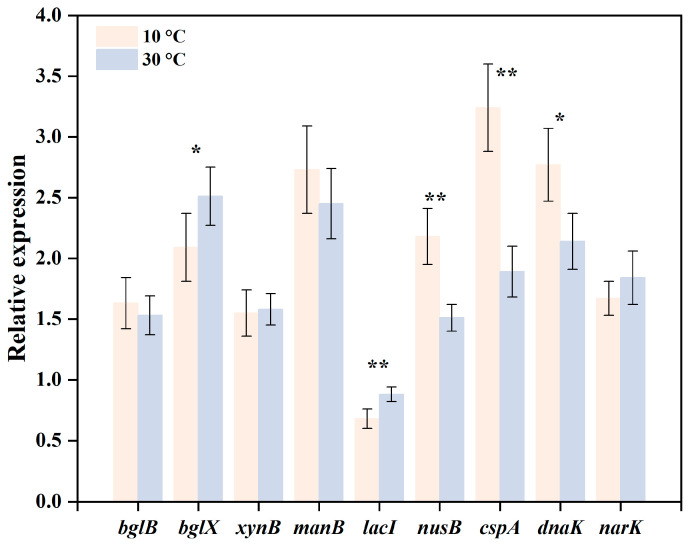
Transcriptional expression profile of functional genes. Data are presented as mean ± SD (*n* = 3). Asterisks indicate significant differences between different temperatures, as determined by Student’s *t*-test. *, *p* < 0.05; **, *p* < 0.01.

To further clarify the molecular basis for the enhanced enzymatic activities and straw degradation performance observed in the mutant strain LDT1-8, the transcriptional responses of representative genes involved in carbohydrate utilization, transcriptional regulation, stress adaptation, and nitrogen transport were examined by qRT-PCR under 10 °C and 30 °C conditions (Figure 8). Compared with the wild-type strain LDT1, the mutant strain exhibited consistently higher transcript levels of multiple CAZyme-related genes, including *bglB*, *bglX*, *xynB*, and *manB*, at both temperatures. Instead of reflecting isolated changes in individual enzymes, this coordinated upregulation suggests that ARTP mutagenesis enhanced the overall transcriptional capacity of the polysaccharide-degrading system. Such coordinated regulation is advantageous for lignocellulose degradation. This process requires the simultaneous action of multiple enzymes acting along interconnected pathways rather than single rate-limiting steps [52]. This transcriptional pattern is consistent with the broadly elevated cellulase and hemicellulase activities and the accelerated straw degradation observed for LDT1-8.

The downregulation of the transcriptional repressor gene *lacI* in the mutant strain provides a plausible regulatory explanation for this coordinated induction. Instead of abolishing repression entirely, the moderate reduction in *lacI* expression observed at both temperatures is indicative of regulatory relaxation rather than loss of control. Similar fine-tuning of transcriptional repression has been shown to promote efficient carbohydrate utilization while preserving regulatory flexibility in other lignocellulose-degrading bacteria [49,53]. In this context, partial alleviation of *lacI*-mediated repression may have lowered the activation threshold for downstream carbohydrate metabolism genes, enabling sustained enzyme production under both low and moderate temperature conditions.

Beyond carbohydrate metabolism, LDT1-8 displayed markedly enhanced transcription of genes associated with transcriptional efficiency and stress adaptation, including *nusB*, *cspA*, and *dnaK*, with particularly strong induction under low-temperature conditions. These genes do not directly participate in polysaccharide hydrolysis but are critical for maintaining cellular information flow and protein homeostasis under thermal stress. Enhanced expression of *nusB* is associated with improved transcriptional processivity and mRNA stability, while *cspA* and *dnaK* contribute to efficient translation and proper protein folding at low temperatures [31,54,55]. The preferential upregulation of these genes at 10 °C suggests that ARTP mutagenesis enhanced not only enzymatic output but also the cellular capacity to sustain transcription, translation, and secretion processes when metabolic reactions are otherwise constrained by cold stress. This systemic reinforcement likely underpins the superior low-temperature performance of LDT1-8.

In addition, the upregulation of the nitrate/nitrite transporter gene *narK* in the mutant strain implies improved nitrogen acquisition and redox balancing during active lignocellulose degradation. Increased nitrogen availability is essential to support the elevated biosynthetic demand associated with enhanced enzyme production, particularly under carbon-rich growth conditions. Similar coupling between nitrogen metabolism and biomass degradation efficiency has been reported in other bacteria adapted to complex organic substrates [56].

Taken together, the combined genomic and transcriptional results provide a plausible mechanistic link between ARTP-induced mutations and the enhanced cellulolytic phenotype observed in LDT1-8. Most of the detected mutations were located in regulatory or intergenic regions, suggesting that they may influence transcriptional control rather than introducing new functional genes. Such regulatory modifications could alter the expression of downstream metabolic pathways involved in carbohydrate utilization. This interpretation is consistent with the coordinated upregulation of multiple CAZyme-related genes observed in the qRT-PCR analysis. The elevated transcription of these genes can promote increased production of cellulolytic and hemicellulolytic enzymes, which corresponds well with the higher enzymatic activities measured in the mutant strain and with the accelerated early-stage straw degradation observed in LDT1-8. Therefore, the improved lignocellulose degradation performance of LDT1-8 is likely associated with mutation-driven regulatory reprogramming that enhances the transcriptional capacity of the cellulolytic enzyme system under low-temperature conditions. Collectively, these findings indicate that ARTP mutagenesis resulted in coordinated transcriptional reprogramming in LDT1-8, enabling more efficient lignocellulose degradation under low-temperature conditions.

## 4. Conclusions

In this study, a cold-tolerant cellulolytic bacterium, *Paenarthrobacter nitroguajacolicus* LDT1, was first identified through functional screening at 10 °C, and its performance was subsequently enhanced by ARTP mutagenesis, yielding the mutant strain LDT1-8. Validation using a straw substrate demonstrated that ARTP mutagenesis primarily strengthened the initiation and early-stage kinetics of straw degradation at low temperatures, rather than merely increasing the final degradation extent. This phenotype was supported by enzyme profiling results showing coordinated enhancement of multiple lignocellulose-degrading activities under low-temperature conditions, together with a partial shift in temperature responsiveness toward colder conditions for selected enzymes. Whole-genome sequencing, comparative genomic analysis, and transcriptional validation revealed that the improved performance of the mutant strain was not due to the acquisition of new functional genes. Instead, it was mainly associated with regulatory mutations and the resulting transcriptional reorganization. Genes involved in cellulose and hemicellulose utilization, cold adaptation, and transcriptional and protein homeostasis processes exhibited coordinated expression changes, collectively supporting more efficient lignocellulose degradation under low-temperature conditions. Overall, this work provides preliminary evidence for the potential application of the mutant strain in low-temperature biomass degradation systems. However, further validation under composting or reactor-scale conditions is required.

## Data Availability

Genome assembly sequence and annotation information have been deposited in the China National Center for Bioinformation database under accession number PRJCA059123. Other supporting data, including qRT-PCR, are available in figshare.

## Supplementary Materials

The following supporting information can be downloaded at https://www.mdpi.com/article/10.3390/microorganisms14040728/s1: Figure S1: Morphological characteristics of LDT1; Figure S2: ARTP mutagenesis time mortality curve; Figure S3: Genetic stability of CMCase activity in strain LDT1-8; Figure S4: The straw degradation rate of strain LDT1-8 under low-temperature condition at different times; Figure S5: CGView comparison of LDT1 and LDT1-8 genome circles; Figure S6: Carbohydrate active enzymes annotation statistics chart; Table S1: Functional characterization of different cellulose-degrading strains under low temperature conditions; Table S2: Identification of physiological and biochemical characteristics of strain LDT1; Table S3: Gene information related to cold tolerance in LDT1; Table S4: Gene information related to cold tolerance in LDT1-8; Table S5: Gene information related to cellulose degradation in LDT1; Table S6: Gene information related to cellulose degradation in LDT1-8; Table S7: Mutation annotation results statistics for LDT1-8; Table S8: Comparative analysis of gene assembly results for LDT1 and LDT1-8 strains; Table S9: Mutation annotation results for LDT1-8; Table S10: The primers used in this study; Supplementary Text S1: Media component; Supplementary Text S2: Determination of Carboxymethyl Cellulase Activity; Supplementary Text S3: genome analysis; Supplementary Text S4: qRT-PCR.
