# Supplementary material for: Mechanisms of Enhanced Low-Temperature Lignocellulose Degradation by an ARTP-Induced Paenarthrobacter nitroguajacolicus Mutant: Physicochemical Characterization, Comparative Genomic Analysis, and Transcriptional Expression Profile Verification
Source: Microorganisms. 2026 Mar 24;14(4):728. doi: 10.3390/microorganisms14040728 (PMC13118209; doi:10.3390/microorganisms14040728)
Supplement: Supplementary file 1 [file microorganisms-14-00728-s001.zip › microorganisms-4174084-supplementary.pdf]

Supplementary text

Text S1 Media component

Cellulose-degrading bacteria screening medium: $(NH_4)_2SO_4$ 2.0 g, $MgSO_4$ 0.5 g, KH_2PO_4 1.0 g, NaCl 0.5 g, sodium carboxymethyl cellulose (CMC-Na) 20.0 g, agar 20.0 g, distilled water 1000 mL; pH adjusted to 6.8–7.2[1].

Filter paper degradation medium: Beef extract 1.5 g, peptone 1.0 g, $CaCO_3$ 0.5 g, distilled water 1000 mL. After adding 7 cm depth of medium into each bottle, a filter paper strip (1 cm × 6 cm) was inserted into each bottle[2].

Straw degradation medium: Corn straw (1 cm × 2 cm pieces) 20 g, $(NH_4)_2SO_4$ 2.0 g, $MgSO_4$ 0.5 g, KH_2PO_4 1.0 g, NaCl 0.5 g, distilled water 1000 mL; pH adjusted to 6.8–7.2[1].

CMC−Congo red agar medium: CMC-Na 20.0 g, $(NH_4)_2SO_4$ 2.0 g, $MgSO_4$ 0.5 g, KH_2PO_4 1.0 g, NaCl 0.5 g, agar 20.0 g, Congo red 0.2 g, distilled water 1000 mL; pH adjusted to 6.8–7.2[2].

Text S2 Determination of Carboxymethyl Cellulase Activity

The reagent kit used in this experiment was purchased from Shanghai Yuan Ye Biotechnology Co., Ltd.

(a) Preparation of enzyme extract solution. Add 10 mL of the enzyme solution to a glass bottle containing sterilized steel beads, then add 20 mL of distilled water for dilution. Shake at 200 r/min for 30 min, and filter through four layers of sterile gauze into a 50 mL centrifuge tube. Centrifuge at 8000 r/min for 10 min, transfer the supernatant to a 50 mL centrifuge tube, add water to make up to the volume, and this is the enzyme extract solution.

(b) Carboxymethyl cellulase determination. Carry out the experiment according to the following parameters:

Additive	CK (mL)	Test (mL)
Enzyme extracting solution	-	0.1
CES Assay buffer	0.1	0.1
CMC Solution (60°C preheating in advance)	0.3	0.3

Incubate at 60°C for 20 min	0.3	0.3
Enzyme extracting solution	0.1	-
Shake well immediately, place in a boiling water bath, allow to color for 5 minutes, then remove immediately and cool under running water.		

Add 200 μL to each tube and place them in the 96-well plate. Zero the "0" tube and measure the absorbance at 540 nm for each tube.

(c) Carboxymethyl cellulase calculation. Using the series of glucose standards (in μg/mL) as the abscissa and the corresponding absorbance as the ordinate, a graph was plotted to obtain the standard curve. Based on this standard curve, the glucose concentration corresponding to the absorbance of the enzyme extract (control tube, test tube) was calculated. Then, according to the formula, the cellulase activity was calculated.

Definition of cellulase activity unit: At 60 °C, 1 mL of enzyme extract, catalyzing the hydrolysis of sodium carboxymethyl cellulose to produce 1 μg of glucose within 1 min, is defined as 1 unit of enzyme activity (U). Based on the definition of enzyme activity, the cellulase activity in the sample was calculated.

$$\mathbf{U} = \frac{\mathbf{k} \times (\mathbf{C_1} - \mathbf{C_0}) \times \mathbf{V_0}}{\mathbf{m} \times \mathbf{t}}$$

K, dilution factor of the sample; C1, glucose concentration of the sample test tube, in units of μg/mL; C_0, glucose concentration of the sample control tube, in units of μg/mL; t, reaction time of the enzyme and substrate, in units of min (= 20); V_0, total volume of the enzyme extraction solution of the solid sample, in units of mL; m, mass of the sample, in units of g (= 10).

Text S3 genome analysis

The whole genome analysis and comparative genomic analysis were all carried out with the assistance of Majorbio Bio-Pharm Technology Co., Ltd. (Shanghai, China).

Library construction and sequencing

Genome sequencing was conducted using a combined method of PacBio RS II single-molecule real-time sequencing (SMRT) and Illumina platform. During the library construction stage, ≥ 1 μg of genomic DNA was selected and cut into fragments of

approximately 400 bp using a Covaris device. The library was prepared using the NEXTflex™ Rapid DNA-Seq kit. Specific fragments were screened by agarose gel electrophoresis, then treated with sodium hydroxide to generate single-stranded DNA, and finally amplified by bridge PCR to ensure the acquisition of high-quality double-stranded DNA libraries.

For the PacBio sequencing library, fragment purification, end repair, and adapter ligation were performed. After three rounds of purification with Agencourt AMPure XP beads, the PacBio library was ligated to the polymerase at the bottom of the ZMW for real-time detection of base synthesis luminescence signals. When sequencing with the Illumina HiSeq X Ten platform in both directions, one base was polymerized per cycle, and the sequence was read using fluorescence labeling, followed by removal of fluorescence and termination groups to proceed to the next cycle of polymerization. The high-resolution optical system ensures the accuracy of data reading, ensuring the quality of the final sequencing data.

The raw data was saved in fastq format. To improve assembly accuracy, quality trimming was performed to remove low-quality, high-N proportion, and short-length reads, resulting in clean data. During the assembly process, Unicycler software was used to assemble contigs based on PacBio data, and manual checks were conducted to determine if rings were formed. Then, the assembly results were corrected using Illumina data to ensure the acquisition of complete chromosome and plasmid genomes.

Mutation prediction and annotation statistics

During the data processing stage, to ensure the quality of the sequencing data, the fastp v0.20.0 software was first used to remove the adapter sequences from the raw data, and a 4 bp sliding window was set to cut off the sequences within the window whose quality values were lower than Q20 (including the sequences at both ends). At the same time, sequences containing N bases were deleted, and trimmed sequences shorter than 30 bp were also removed. Next, the BWA software (http://bio-bwa.sourceforge.net/) with the mem algorithm was used to align the obtained reads with the reference genome. Based on the BAM file generated by the alignment, the sequencing depth and coverage were

measured. Subsequently, the GATK software was used to generate a BAM file after realignment processing. Then, the snippy 4.6.0 software was used to detect SNPs and small InDels, and the variant sites were selected. Finally, with the help of snpEff (http://snpeff.sourceforge.net/SnpEff.htm), the detected variant sites were annotated to assess the potential impact of these mutations on the genome function.

Text S4 qRT-PCR

Strains LDT1 and LDT1-8 were inoculated (2%, v/v) into liquid medium containing CMC-Na as the sole carbon source and incubated at 10 °C and 30 °C with shaking at 200 rpm. Cells were harvested at the exponential growth phase by centrifugation, immediately frozen in liquid nitrogen, and stored at −80 °C. Total RNA was extracted using the MiniBEST Bacteria RNA Extraction Kit (TaKaRa, Japan) and treated with DNase I to remove residual genomic DNA. RNA quality and concentration were determined using a NanoDrop 2000 spectrophotometer, and samples with A260/A280 ratios between 1.8 and 2.1 were used for subsequent analysis. First-strand cDNA was synthesized from equal amounts of RNA using the PrimeScript™ RT Reagent Kit (TaKaRa, Japan). Gene-specific primers were designed based on the genome sequences of LDT1 and LDT1-8 to quantify genes involved in cellulose and hemicellulose degradation (*bglB*, *bglX*, *xynB*, *manB*), transcriptional regulation (*lacI*, *nusB*), cold adaptation and molecular chaperones (*cspA*, *dnaK*), and nitrogen metabolism (*narK*). All primers were synthesized by Sangon Biotech (Shanghai, China) and listed in Table S10. The 16S rRNA gene was used as the internal reference for normalization. Relative gene expression levels were calculated using the $2^{-\Delta\Delta Ct}$ method, with the wild-type strain LDT1 at the corresponding temperature used as the calibrator.

Reference

1. Feng H W. Study on the degradation of lignocellulose and nitrate conversion by Streptomyces cinereus in compost[D]. Shanghai: Shanghai Jiaotong University, 2015.
2. Feng H W, Zhou P, Mao L, et al. Screening of an efficient cellulose degrading strain and optimization of enzyme production conditions[J]. Journal of Shanghai Jiaotong

University (Agricultural Science Edition), 2013, 31(02): 24-9.

Supplementary figures

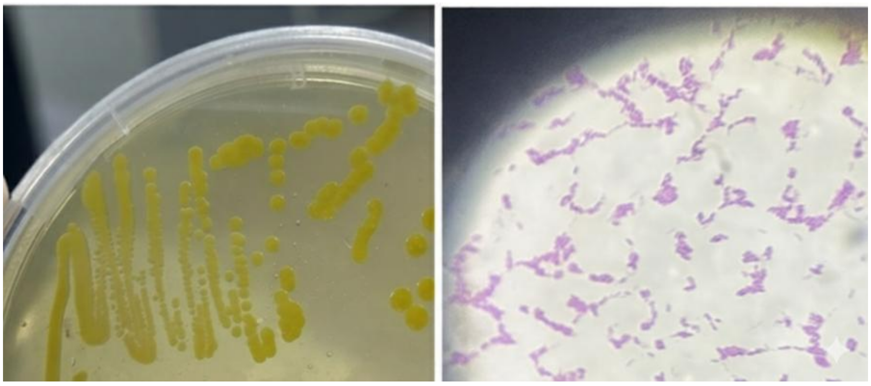

Fig. S1 Morphological characteristics of LDT1.

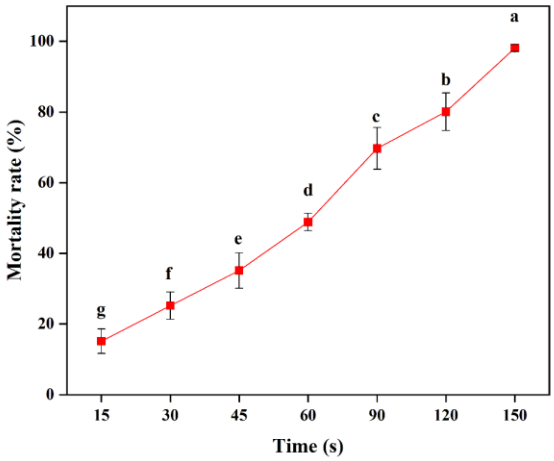

Fig. S2 ARTP mutagenesis time mortality curve. Data are presented as mean ± SD (n = 3). Different letters represent significant differences between groups according to Tukey's multiple comparison ($P < 0.05$).

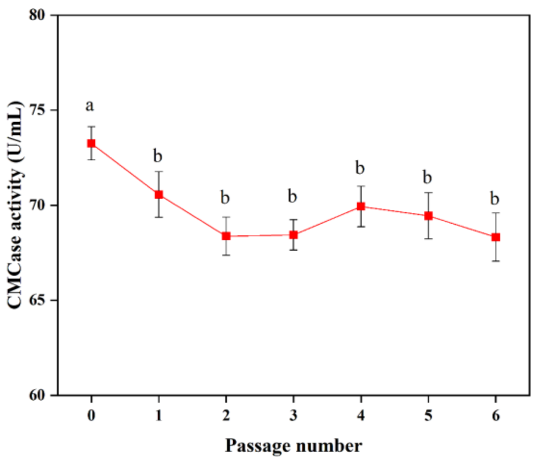

Fig. S3 Genetic stability of CMCase activity in strain LDT1-8. Data are presented as mean ± SD (n = 3). Different letters represent significant differences between groups according to Tukey's multiple comparison ($P < 0.05$).

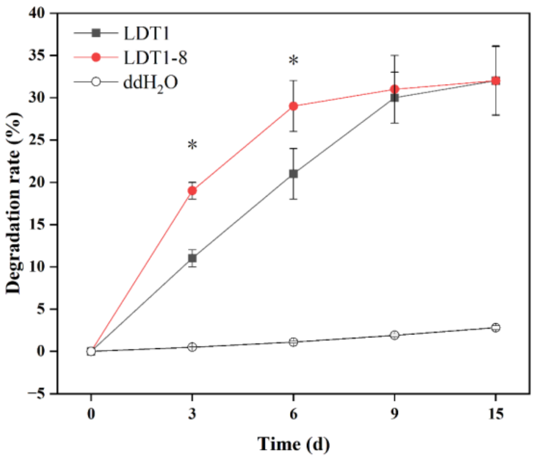

Fig. S4 The straw degradation rate of strain LDT1-8 under low-temperature condition at different times. Data are presented as mean ± SD (n = 3). Asterisks indicate significant differences compared to the LDT1 group, as determined by student's *t*-test ($P < 0.05$).

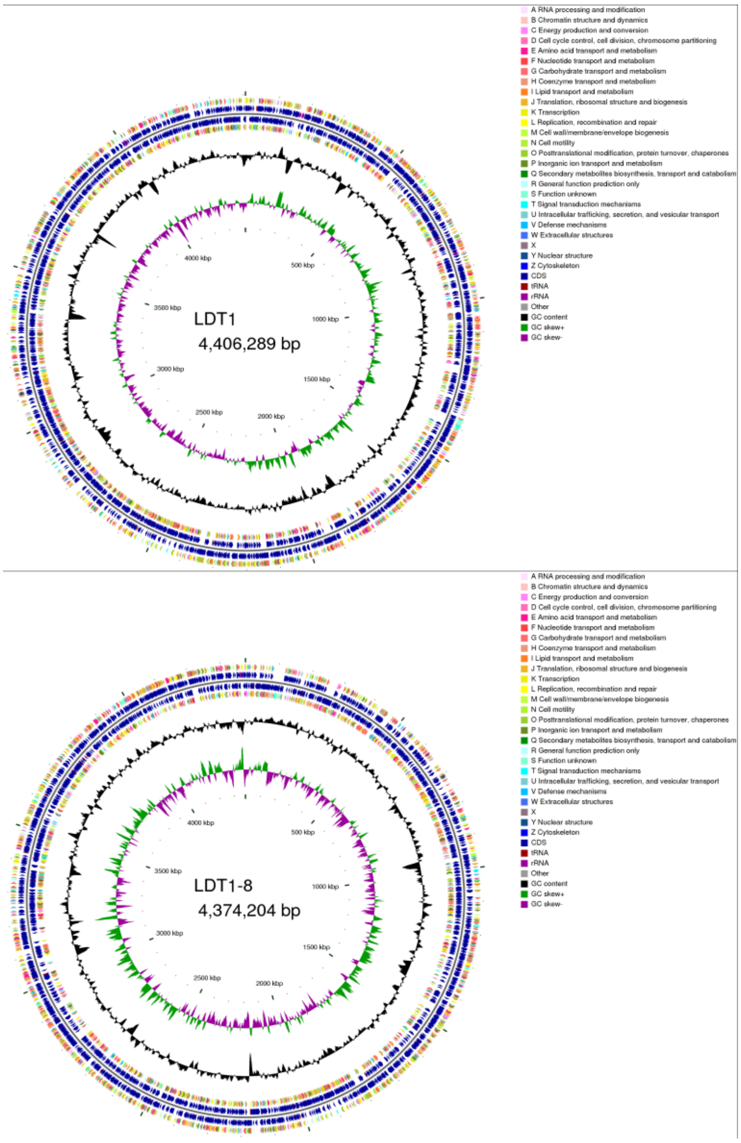

Fig. S5 CGView comparison of LDT1 and LDT1-8 genome circles

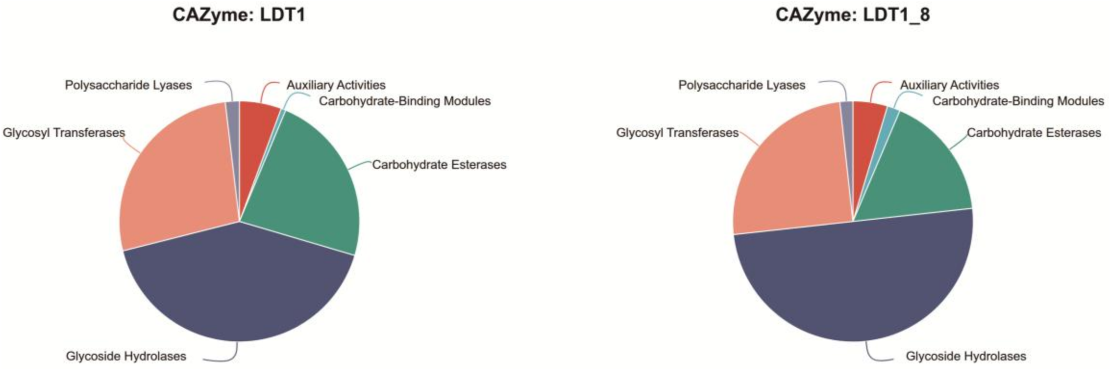

Fig. S6 Carbohydrate active enzymes annotation statistics chart

Supplementary tables

Table S1 Functional characterization of different cellulose-degrading strains under low temperature conditions

Strain ID	Relative activity	Filter paper degradation	Straw degradation
LDT1	1.16	++	++
LDS2	-	+	+++
LDT5	-	++	++
LDT2	-	+	++
LTO6	-	++	+
LTS5	-	++	+
LDF4	1.29	++	+
LTO7	-	+	++
LDF2	-	+	+
LTO5	1.09	++	+++
LTO9	1.03	+	+
LDF6	1.65	++	+
LDF5	1.43	++	++
LDT3	1.02	++	+
LTS6	1.04	++	+++
LTF5	1.31	++	+
LTO10	1.04	+++	+
LTS1	-	+	++
LTF2	-	+	+
LDS8	-	++	++
LDS6	1.01	+	+++
LTS3	1.00	++	++
LTS2	-	++	+++
LDS7	-	++	+

Note: "-" indicates the absence of hydrolysis rings; "+" indicates slight degradation of straw or filter paper; "++" indicates straw or filter paper degradation with the appearance of turbidity; "+++"

indicates that the straw or filter paper was largely degraded.

Table S2 Identification of physiological and biochemical characteristics of strain LDT1

Test item	LDT1	Test item	LDT1
Hydrogen sulfide (H_2S)	–	Glucose	+
Phenylalanine	–	Sucrose	–
Gluconate	–	Lactose	–
Peptone water	+	Arabinose	–
Phosphate peptone water	–	Maltose	–
Citrate utilization	+	Nitrate reduction	+
Urease	+	Nitrate oxidation	–
Semi-solid motility test	+	Potassium cyanide (KCN)	–
Gas production	+	Arginine dihydrolase	–
Lysine decarboxylase	+	Galactose broth	–
Ornithine decarboxylase	+	Arginine decarboxylase	+
Gossypol	+	Sodium pyruvate	–
Sorbitol	+	Mannitol	–
Marigold alcohol	+	Alanine	–
Xylose	–	Inositol	–
Malonate	–	Hugh-Leifson oxidation	–

Note: +, positive; -, negative.

Table S3 Gene information related to cold tolerance in LDT1

Sample Name	Gene ID	Gene Name	Start	End	Length (bp)
LDT1	gene0190	*clpC*	190841	193333	2493
LDT1	gene0859	*clpC*	880981	881433	453
LDT1	gene0896	*trxB*	913384	914385	1002
LDT1	gene0914	*cspA*	933919	934122	204
LDT1	gene0915	*groEL*	934471	936096	1626
LDT1	gene0928	*cspA*	949568	949951	384
LDT1	gene1089	*cspA*	1123211	1123414	204
LDT1	gene1101	*dnaJ*	1137595	1137984	390
LDT1	gene1480	*recA*	1561995	1563038	1044

LDT1	gene1652	*cydA*	1751307	1752905	1599
LDT1	gene1653	*cydB*	1752924	1754006	1083
LDT1	gene1872	*trxB*	1990703	1989741	963
LDT1	gene1927	*katE*	2044086	2046308	2223
LDT1	gene1935	*trxB*	2057091	2055352	1740
LDT1	gene1937	*dnaK*	2057847	2059745	1899
LDT1	gene1941	*clpB*	2061541	2064177	2637
LDT1	gene2024	*cspA*	2154813	2154610	204
LDT1	gene2251	*dnaJ*	2404919	2403792	1128
LDT1	gene2252	*hrcA*	2405977	2404970	1008
LDT1	gene2306	*ppiB*	2464686	2465480	795
LDT1	gene2596	*cspA*	2768204	2768407	204
LDT1	gene2600	*atpC*	2773113	2772829	285
LDT1	gene2601	*atpD*	2774566	2773115	1452
LDT1	gene2602	*atpG*	2775541	2774651	891
LDT1	gene2603	*atpA*	2777288	2775618	1671
LDT1	gene2604	*atpH*	2778167	2777340	828
LDT1	gene2605	*atpF*	2778724	2778167	558
LDT1	gene2606	*atpE*	2778993	2778775	219
LDT1	gene2607	*atpB*	2779874	2779074	801
LDT1	gene2608	*atpI*	2780291	2779956	336
LDT1	gene2609	*atpI*	2780769	2780278	492
LDT1	gene2875	*groEL*	3078919	3077306	1614
LDT1	gene2876	*groES*	3079300	3079004	297
LDT1	gene3044	*trxA*	3246432	3246055	378
LDT1	*gene3062*	*katE*	3261682	3263163	1482
LDT1	gene0190	*clpC*	3490789	3488687	2103
LDT1	gene0859	*clpC*	3743107	3740450	2658
LDT1	gene0896	*trxB*	3806199	3805216	984
LDT1	gene0914	*cspA*	3808904	3807039	1866
LDT1	gene0915	*groEL*	3832206	3831229	978
LDT1	gene0928	*cspA*	4392648	4393604	957
LDT1	gene1089	*cspA*	4393639	4393965	327

Table S4 Gene information related to cold tolerance in LDT1-8

Sample Name	Gene ID	Gene Name	Start	End	Length (bp)
LDT1_8	gene0323	*cspA*	368609	368406	204
LDT1_8	gene0311	*dnaJ*	354420	353836	585
LDT1_8	gene0498	*cspA*	557901	557698	204
LDT1_8	gene0608	*groES*	30923	30627	297
LDT1_8	gene0607	*groEL*	30542	28929	1614
LDT1_8	gene0484	*cspA*	542252	541869	384
LDT1_8	gene0497	*groEL*	557349	555724	1626

LDT1_8	gene0553	*clpC*	610839	610387	453
LDT1_8	gene0516	*trxB*	578436	577435	1002
LDT1_8	gene0794	*katE*	213305	214786	1482
LDT1_8	gene0776	*trxA*	198055	197678	378
LDT1_8	gene0998	*dnaK*	443041	440939	2103
LDT1_8	gene1337	*atpI*	267519	267854	336
LDT1_8	gene1340	*atpF*	269086	269643	558
LDT1_8	gene1339	*atpE*	268817	269035	219
LDT1_8	gene1344	*atpD*	273244	274695	1452
LDT1_8	gene1343	*atpG*	272269	273159	891
LDT1_8	gene1338	*atpB*	267936	268736	801
LDT1_8	gene1342	*atpA*	270522	272192	1671
LDT1_8	gene1345	*atpC*	274697	274981	285
LDT1_8	gene1336	*atpI*	267041	267532	492
LDT1_8	gene1341	*atpH*	269643	270470	828
LDT1_8	gene1349	*cspA*	279606	279403	204
LDT1_8	gene1708	*hrcA*	225565	224558	1008
LDT1_8	gene1762	*ppiB*	284274	285068	795
LDT1_8	gene1707	*dnaJ*	224507	223380	1128
LDT1_8	gene2036	*clpB*	139909	137252	2658
LDT1_8	gene2121	*trxB*	229008	228031	978
LDT1_8	gene2097	*dnaJ*	203001	202018	984
LDT1_8	gene2099	*dnaK*	205706	203841	1866
LDT1_8	gene2288	*clpC*	6515	9007	2493
LDT1_8	gene2793	*cydA*	122510	124108	1599
LDT1_8	gene2794	*cydB*	124127	125209	1083
LDT1_8	gene3012	*trxB*	361981	361019	963
LDT1_8	gene3176	*trxA*	154992	155318	327
LDT1_8	gene3175	*trxB*	154001	154957	957
LDT1_8	gene3874	*recA*	68702	69745	1044
LDT1_8	gene4054	*katE*	37411	35189	2223
LDT1_8	gene4046	*trxB*	24406	26145	1740
LDT1_8	gene4040	*clpB*	19956	17320	2637
LDT1_8	gene4044	*dnaK*	23650	21752	1899
LDT1_8	gene3998	*cspA*	72863	72660	204

Table S5 Gene information related to cellulose degradation in LDT1

Sample Name	Gene ID	Gene Name	Start	End	Length (bp)
LDT1	gene0100	*bglB*	95345	93888	1458
LDT1	gene0221	*abfA*	220842	222368	1527
LDT1	gene0625	*bglX*	627628	625133	2496
LDT1	gene0653	*abfA*	657276	658814	1539

LDT1	gene0731	*bglX*	734896	736158	1263
LDT1	gene0777	*manB*	785064	783646	1419
LDT1	gene1002	*xylA*	1040278	1038794	1485
LDT1	gene1220	*manA*	1262465	1261215	1251
LDT1	gene1289	*manB*	1343647	1341902	1746
LDT1	gene1767	*xynB*	1876188	1874677	1512
LDT1	gene1768	*bglB*	1877502	1876288	1215
LDT1	gene1777	*bglX*	1889334	1886866	2469
LDT1	gene1778	*xynB*	1890328	1889450	879
LDT1	gene1801	*xynD*	1921314	1922450	1137
LDT1	gene1927	*katE*	2044086	2046308	2223
LDT1	gene1939	*cbpA*	2060280	2061230	951
LDT1	gene1947	*bglX*	2068025	2070280	2256
LDT1	gene2950	*bglB*	3144505	3145938	1434
LDT1	gene3062	*katE*	3261682	3263163	1482
LDT1	gene3110	*bglX*	3320292	3322163	1872
LDT1	gene3303	*gdhA*	3533934	3532597	1338
LDT1	gene3673	*xylA*	3915453	3916640	1188
LDT1	gene3674	*xylB*	3916728	3918158	1431

Table S6 Gene information related to cellulose degradation in LDT1-8

Sample Name	Gene ID	Gene Name	Start	End	Length (bp)
LDT1_8	gene0192	*manA*	229356	230606	1251
LDT1_8	gene0122	*manB*	148174	149919	1746
LDT1_8	gene0410	*xylA*	451542	453026	1485
LDT1_8	gene0794	*katE*	213305	214786	1482
LDT1_8	gene0682	*bglB*	96128	97561	1434
LDT1_8	gene1036	*gdhA*	486186	484849	1338
LDT1_8	gene0842	*bglX*	271915	273786	1872
LDT1_8	gene2205	*xylB*	313530	314960	1431
LDT1_8	gene2204	*xylA*	312255	313442	1188
LDT1_8	gene2319	*abfA*	36516	38042	1527
LDT1_8	gene2908	*xynB*	247391	245880	1512
LDT1_8	gene2918	*bglX*	260537	258069	2469
LDT1_8	gene2909	*bglB*	248705	247491	1215
LDT1_8	gene2919	*xynB*	261531	260653	879
LDT1_8	gene2942	*xynD*	292517	293653	1137
LDT1_8	gene3286	*bglB*	262987	261530	1458
LDT1_8	gene3405	*bglX*	47741	45246	2496
LDT1_8	gene3510	*bglX*	154204	155466	1263
LDT1_8	gene3556	*manB*	204372	202954	1419
LDT1_8	gene3433	*abfA*	77389	78927	1539

LDT1_8	gene4054	*katE*	37411	35189	2223
LDT1_8	gene4034	*bglX*	13472	11217	2256
LDT1_8	gene4042	*cbpA*	21217	20267	951

Table S7 Mutation annotation results statistics for LDT1-8

Region	LDT1-8
DOWNSTREAM	11
UPSTREAM	12
INTERGENIC	1
EXON	2

Table S8 Comparative analysis of gene assembly results for LDT1 and LDT1-8 strains

Index	LDT1	LDT1-8
Chromosome No.	1	-
Plasmid No.	0	-
Total Bases in Scaf (bp)	4406289	4374204
Total Scaf No.	-	25
Large Scaf No.	-	21
Large Scaf Bases(bp)	-	4371358
Largest Scaf Len(bp)	-	639197
Scaf N50(bp)	-	401342
Scaf N90(bp)	-	192112
G+C(%)	61.96	62
N Rate(%)	-	0.003
Total Ctg No.	-	39
Total Bases in Ctg (bp)	-	4374072
Large Ctg No.	-	32
Large Ctg Bases(bp)	-	4370404
Largest Ctg Len(bp)	-	639197
Ctg N50(bp)	-	392306
Ctg N90(bp)	-	83428
Depth	-	278.34

Completeness(%)	99.5	99.2

Table S9 Mutation annotation results for LDT1-8

Num	Region Type	gene name
1	upstream_gene_variant	*nasC*
2	upstream_gene_variant	*narK*
3	downstream_gene_variant	*shiA*
4	downstream_gene_variant	*bccA*
5	downstream_gene_variant	*yhdE*
6	downstream_gene_variant	*CDS_Chromosome_1360546_1363065*
7	intergenic_region	*shiA-bccA*
8	missense_variant	*aroE*
9	upstream_gene_variant	*nusB*
10	upstream_gene_variant	*efp*
11	upstream_gene_variant	*CDS_Chromosome_2447273_2447794*
12	upstream_gene_variant	*aroB*
13	upstream_gene_variant	*aroK*
14	upstream_gene_variant	*aroC*
15	downstream_gene_variant	*prsW*
16	downstream_gene_variant	*CDS_Chromosome_2451513_2453195*
17	downstream_gene_variant	*ruvX*
18	downstream_gene_variant	*alaS*
19	missense_variant	*lacI*
20	upstream_gene_variant	*yesO*
21	upstream_gene_variant	*yesQ*
22	upstream_gene_variant	*yesP*
23	upstream_gene_variant	*pepQ*
24	downstream_gene_variant	*CDS_Chromosome_3518721_3520019*
25	downstream_gene_variant	*aldH*
26	downstream_gene_variant	*aci*

Table S10 The primers used in this study.

Gene	F	R

bglB	TGGGAATCACGCTCAACCTC	ACCCTGTTCTGCAGCGAATC
bglX	AAGGTGGCCGAGGGCATCC	GTCTTCACCGGAGATCTCC
xynB	TCCAAGCATCGTCCGCGTC	ACGTTGCCAATCTGCTCCC
manB	TCGACATCGACCACATGGAC	CCTGATCCTTGGCTTCTGCG
lacI	CCCTTGGATGAGTCCTTGG	CATGTCGTCCGTGCAGAAG
nusB	TCCTCCGAATCGGTGCGTG	TGATGAAGGCCGGTGACTC
cspA	ATGGCTTTGGGAACCGTCAA	GTGCACAAAGACGTCGTCTC
dnaK	AGAACTGATTTCCGAGCTCC	CAGAAGTCCCAGGCTGAGAC
narK	GTTAACTTGCCAATGGCGG	AGGTGCGGTTCTTTGAGGG
16S RNA	AATACGTTCCCGGGCCTTG	TAGTCCCAATCGCCGGTCC
